# Light traps fail to estimate reliable malaria mosquito biting rates on Bioko Island, Equatorial Guinea

**DOI:** 10.1186/1475-2875-11-56

**Published:** 2012-02-24

**Authors:** Hans J Overgaard, Solve Sæbø, Michael R Reddy, Vamsi P Reddy, Simon Abaga, Abrahan Matias, Michel A Slotman

**Affiliations:** 1 Department of Mathematical Sciences and Technology, Norwegian University of Life Sciences, Ås, Norway; 2 Department of Chemistry, Biotechnology and Food Science, Norwegian University of Life Sciences, Ås, Norway; 3 Department of Epidemiology and Public Health, Yale University, New Haven, CT, USA; 4 Department of Entomology, Texas A&M University, College Station, TX, USA; 5 National Malaria Control Program, Ministry of Health and Social Welfare, Malabo, Equatorial Guinea; 6 Medical Care Development International, Malabo, Equatorial Guinea

## Abstract

**Background:**

The human biting rate (HBR), an important parameter for assessing malaria transmission and evaluating vector control interventions, is commonly estimated by human landing collections (HLC). Although intense efforts have been made to find alternative non-exposure mosquito collection methods, HLC remains the standard for providing reliable and consistent HBRs. The aim of this study was to assess the relationship between human landing and light trap collections (LTC), in an attempt to estimate operationally feasible conversion factors between the two. The study was conducted as part of the operational research component of the Bioko Island Malaria Control Project (BIMCP), Equatorial Guinea.

**Methods:**

Malaria mosquitoes were collected indoors and outdoors by HLCs and LTCs in three villages on Bioko Island, Equatorial Guinea during five bimonthly collections in 2009. Indoor light traps were suspended adjacent to occupied long-lasting, insecticide-treated bed nets. Outdoor light traps were placed close to the outer wall under the roof of the collection house. Collected specimens were subjected to DNA extraction and diagnostic PCR to identify species within the *Anopheles gambiae *complex. Data were analysed by simple regression of log-transformed values and by Bayesian regression analysis.

**Results:**

There was a poor correlation between the two collection methods. Results varied by location, venue, month, house, but also by the statistical method used. The more robust Bayesian analyses indicated non-linear relationships and relative sampling efficiencies being density dependent for the indoor collections, implying that straight-forward and simple conversion factors could not be calculated for any of the locations. Outdoor LTC:HLC relationships were weak, but could be estimated at 0.10 and 0.07 for each of two locations.

**Conclusions:**

Light trap collections in combination with bed nets are not recommended as a reliable method to assess human biting rates on Bioko Island. Different statistical analyses methods give variable and inconsistent results. Substantial variation in collection methods prevents the determination of reliable and operationally feasible conversion factors for both indoor and outdoor data. Until improved mosquito collection methods are developed that can provide reliable and unbiased HBR estimates, HLCs should continue to serve as the reference method for HBR estimation.

## Background

Assessing the success of malaria vector control interventions requires a robust and accurate entomological monitoring system. To assess if vector control interventions have an impact on malaria transmission, the human biting rate (HBR), i.e. the density of mosquitoes engaged in blood feeding, is an essential parameter. The HBR is a function of overall mosquito density, propensity to bite humans, and frequency of feeding. The product of the HBR and the sporozoite rate, i.e. the percentage of infectious mosquitoes, provides the entomological inoculation rate (EIR). The EIR is the average number of infectious mosquito bites a person receives per time unit, a measure that best represents transmission intensity [1-3]. The widely accepted standard for estimating the HBR is the human landing collection method (HLC).

Human landing collections are typically conducted by volunteers trained to collect host-seeking mosquitoes that land on exposed body parts during the evening and night hours when anopheline vectors are most active. However, HLCs are logistically difficult and expensive to carry out, because volunteers need to be recruited and trained; continuous supervision is essential to avoid loss and attrition of volunteers throughout the night; sorting and identification of collected non-target species is time-consuming. These are all issues that impact the quality of HLCs to estimate HBR and increase the cost per mosquito collected. The results are not always reliable and consistent, due to differential attractiveness of individual collectors to mosquitoes, fatigue and ineffectiveness and/or misconduct of collectors. Furthermore, HLC is considered unethical by some, because collectors are exposed to potentially lethal mosquito bites [4]. On the other hand, even though volunteers are intentionally exposed to potential bites during HLCs, they would be exposed anyhow, given they live in endemic areas. Further, it could be argued that proper training of volunteers results in increased vigilance to host-seeking mosquitoes and therefore may in fact have a protective effect.

The World Health Organization recommends avoiding HLC unless absolutely essential, especially if safer techniques are available that can provide proxy estimates of human biting rates [5]. Despite numerous attempts to find equivalent sampling methods, however, HLC prevails as the only reliable method to determine HBR [6-8]. Light traps (LT), window exit traps, indoor pyrethrum knockdown catches, and outdoor pit traps do not provide a direct estimate of the HBR because they do not specifically capture mosquitoes engaged in host-seeking.

Light traps in combination with occupied mosquito bed nets, where a person under the net functions as a mosquito attractant, have been proposed as a comparable and unbiased alternative to HLC in sampling blood meal-seeking mosquitoes [9]. Many studies have been conducted in Africa and elsewhere, to assess how well light traps provide reliable estimates of human biting rates compared to other methods [7,9-22]. In Africa, the ratio of the number of *Anopheles gambiae *s.l. collected by indoor LTs *versus *indoor HLC varied between 1.06-1.91. However these numbers are based on varying ratios of light traps and human collectors (LTC:HLC); such as 3:2 [9,12], 1:2 [10], or 1:1 [11,13], and cannot,therefore, be compared directly. After adjusting for the varying number of light traps and human collectors, the one-to-one LTC:HLC ratio for *An. gambiae *s.l. ranged from 0.59 in Bagamoyo, Tanzania [10], 1.08 in Burkina Faso [11], 1.59 in Sierra Leone [12], 1.6 in Muheza, Tanzania [9], to 1.91 in Zambia [13]. The two Tanzanian ratios differed by 2.7-fold even though the collections were carried out close in time (five to seven years) and space (140 km). The density of mosquitoes was not reported as having any effect on the relative sampling efficiency in these studies.

Several studies have compared various types of human-baited tent trap designs with LTCs and HLCs for estimating relative sampling efficiencies [15-19]. These studies also report widely varying 1:1 LTC:HLC relative sampling efficiencies; 0.02 in urban Dar es Salaam, Tanzania [19]; 0.33 in the Kilombero valley, Tanzania [17]; 0.56 in Ahero, western Kenya [16]; 1.3 also in Kilombero, Tanzania [18]; and 1.86 in Lwanda, western Kenya [15]. It was suggested that the extremely low efficiency of light traps in Dar es Salaam was caused by the highly illuminated, urban environment reducing the efficacy of the light source in the traps [19]. The large difference between the two Kenyan studies was explained by the use of bed nets that repelled the mainly zoophilic *Anopheles arabiensis *to seek alternative hosts outdoors and differences due to the presence/absence of cattle between sites [11,12]. The reason for the even larger differences between the Tanzanian studies [17,18], conducted in the same village just a few years apart, were suggested to be effects of minor, uncontrollable factors, such as location, vector control interventions, season, weather and house type, all of which may vary through space and time.

Light traps have generally been found to underestimate the abundance of host-seeking anophelines [20-22]. Along the Kenyan coast, light traps performed poorly at lower densities and the sporozoite rate was significantly higher in mosquitoes collected using light traps compared to human landing collections [21]. On this basis, it was recommended that light traps should not be used as a substitute to human landing collections in areas with low malaria mosquito densities. However, Smith [23] emphasized that the log (x + 1) transformation of data, commonly used in trap comparison studies [e.g. [9,21]], is highly dependent on mosquito density, particularly at low values of x. Therefore this transformation may not be valid in areas with sparse mosquito counts, such as in Kenya. Poisson regression techniques would be more appropriate for such analyses, because equivocal data transformations are avoided and disproportionally influential low mosquito counts are managed by weighting low density observations [23]. Consequently, Hii *et al*. [14] in Papua New Guinea used a novel statistical approach based on parameterizing the negative binomial as a gamma mixture of Poisson distributions to model agreement between sampling methods, assuming both proportionality and non-linear relationships. These results showed that light traps underestimated the abundance of *Anopheles punctulatus *and *Anopheles farauti *s.l. at high densities. On the other hand, the LTC:HLC ratio for *Anopheles koliensis *and *Anopheles karwari *increased with increasing mosquito density. The authors concluded that light traps could not be calibrated to give reliable estimates of HBR in Papua New Guinea [14].

The present study was conducted as part of the operational research component of the Bioko Island Malaria Control Project (BIMCP) to determine if LTCs can be used to accurately estimate HBR on Bioko Island, Equatorial Guinea. Bioko Island is characterized as a humid tropical environment with hyper-endemic malaria transmission, high mosquito densities, and high entomological inoculation rates. Indoor and outdoor LTCs were analysed and compared with HLCs at several locations and time points to explore the consistency of LTC:HLC ratios for Bioko Island.

## Methods

### Study area

**Figure 1:**
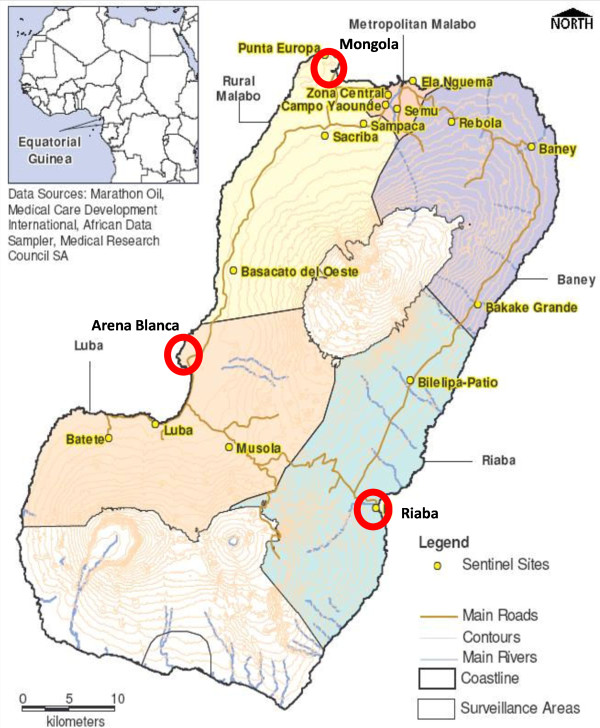
**Map of study villages on Bioko Island, Equatorial Guinea**. Red circles show study sites: Mongola, Arena Blanca, and Riaba. Yellow-shaded names are sentinel sites of the Bioko Island Malaria Control Project (BIMCP), Bioko Island, Equatorial Guinea.

Bioko Island, Equatorial Guinea is located in the Bay of Guinea in Central Africa (N 3° 40', E 8° 50'). The mean annual rainfall is ~2,000 mm/yr. Generally, the rainy season starts in May and ends in October. Mean daily maximum and minimum temperatures range between 29-32°C and 19-22°C, respectively. This study was carried out in three villages on Bioko Island: Mongola, Arena Blanca, and Riaba (Figure 1). These three sites are included in a set of 17 sentinel sites in which routine entomological monitoring and annual parasitaemia surveys are carried out within the Bioko Island Malaria Control Project (BIMCP 2004-2013). All sites have access to electric power, but are poorly illuminated during the night. In Arena Blanca and Riaba there are no street lights. However, Mongola is situated in an industrial area close to a larger road with street lights and the international airport and is, therefore, more lit up than the other sites.

### Mosquito collections

Mosquitoes were collected by human landing collections (HLC) and light trap collections (LTC) in March, May, July, September, and November 2009. Collections were carried out during two consecutive days, except in March in Mongola when four days of consecutive collections were undertaken. In each site, four pairs of houses were randomly selected from a list of houses used in the annual BIMCP parasitaemia survey. Where feasible, the same houses were used throughout the study. The two collection methods were alternated each night, i.e. in a house where HLC was undertaken during the first night, LTC was performed the next night and *vice versa*.

In a house assigned to HLC, two local volunteers, one indoors and the other outdoors, collected mosquitoes landing on exposed legs and feet from 19:00 to 06:00, with a five-minute break each hour. Indoor and outdoor collectors changed venues at midnight. Collectors were recruited from each of the communities. The HLC protocol of BIMCP provides free diagnosis and treatment to any collector who develops symptoms. The collected specimens were separated by hour. At a house assigned to LTC, two modified CDC light traps fitted with ultraviolet light emitting diodes (UV LED, wave length: 385-395 nm) [24] were installed; one indoors and one outdoors. Indoor light traps were suspended approximately 0.2-0.3 m from an occupied long-lasting insecticide-treated bed net (LLIN) (Permanet^® ^2.0, Vestergaard Frandsen), generally at the foot end of the bed 1.5 m from the floor. The rationale for using insecticide-treated nets, and not untreated nets, was to calculate functional and practical field-based LTC:HLC conversion factors, since LLINs are a general component of the BIMCP and bed nets were provided to communities during an intensive mass distribution campaign in 2007 and most nets in use were obtained during that campaign. In case nets were broken or absent, new nets were provided. The outdoor light trap was placed under the roof at 0.2-0.3 m from the outer wall, approximately 1.5 m from the ground. The outdoor light trap was hung on the opposite side of the house from the room where the indoor light trap was placed. All light traps operated from 18:00 to 06:00 hours. Thus, on each collection night, eight human collectors (four outdoors and four indoors) and eight light traps (four outdoors and four indoors) operated at each site. Consequently, a total of 128 indoor collection nights and 128 outdoor collection nights were conducted for each method in all sites during the whole study period.

Ethical approval for this study was granted by the National Malaria Control Programme (NMCP) of the Ministry of Health and Social Welfare, Equatorial Guinea. The lead entomologist (SA) of the NMCP was present and provided supervisory support for all collection activities.

### Molecular analyses

Collected mosquitoes were brought back to the laboratory; sorted, counted, and classified according to genus and blood-feeding status (unfed or fed, i.e. visible traces of blood in abdomens) during each collection event. Specimens were further sorted by collection method, day and hour, placed in 70-95% ethyl alcohol, and stored until molecular analyses were performed. Heads and thoraces were dissected and subjected to DNA extraction using a QIAGEN Biosprint (QIAGEN Sciences Inc., Germantown, MD). A diagnostic PCR followed by restriction enzyme digest was used for species identification within the *An. gambiae *s.l. complex [25].

### Statistical analysis

The total nightly indoor number of mosquitoes in the LTC was compared with those of the HLC, in all sites together and in each site separately, by a simple linear regression analysis on log-transformed (logx + 1) values [26]. To compare methods and test if the relative sampling efficiency was affected by mosquito density, the ratio of the number of mosquitoes in LTC to the number of mosquitoes in HLC (log(HLC + 1) - log(LTC + 1)) was plotted against the average abundance, [log(HLC + 1) + log(LTC + 1)]/2 [26]. These analyses were done using SPSS 16.0 statistical software [27]. Due to the reported statistical weaknesses of adding one to the counts [23], a more rigorous regression-based analysis was performed using a slight modification of the approach suggested by Hii *et al*. [14]. The main difference was that other factors, i.e. month and house, were included in the model for the expected counts of HLC. Separate analyses were run for each of the three locations and also with regard to venue (indoors or outdoors). For a given location and venue let *y*_*ijk *_denote the *k*^th ^observed LTC count in month *i *(*1 = March, 2 = May, 3 = July, 4 = September, 5 = November*) and in house *j *(*j = 1,2,3,4*). Further let *x*_*ijk *_be the corresponding count using HLC. As in Hii *et al*. [14], it was assumed that both *y*_*ijk *_and *x*_*ijk *_are Poisson distributed, hence:

$$y_{ijk}\sim Poisson(\lambda_{ijk})$$

$$x_{ijk}\sim Poisson(\kappa_{ijk})$$

Further the expectation parameter *λ*_*ijk *_of *y*_*ijk *_is taken to be either linearly (model 1) or nonlinearly (model 2) related to the expected HLC count *κ*_*ijk *_as follows:

$$\text{Model 1}:\lambda_{ijk}=\beta_{0}\kappa_{ijk}$$

$$\text{Model 2}:\lambda_{ijk}=\beta_{0}\kappa_{{}_{ijk}}^{\beta_{1}}$$

Model 1 reflects that the expected counts from the two collection methods are proportional, whereas model 2 reflects a density dependent relation between LTC and HLC. Further, it is assumed that the expected count of *x*_*ijk *_depends log-linearly on both month and house:

$$\log(\kappa_{ijk})=\mu +\theta_{i}+\gamma_{j}$$

where *θ*_*i *_is the additional effect of month *i *and *γ*_*j *_is additional the effect of house *j *to the general level *μ*. The expected counts of LTC correspondingly depend on the effects of month and house through this expression for the expected HLC counts. A Bayesian approach using Markov Chain Monte Carlo methods (MCMC) e.g. [28], was adopted for parameter estimation. The models were implemented in WinBUGS (Windows version of Bayesian Updating using Gibbs Sampling) with wide normal priors for all parameters except *β*_0 _for which a wide lognormal prior was assumed to ensure positive expected value of *y*_*ijk*_. Parameter estimates were obtained as the means of the sampled posterior distribution of each parameter.

The model fits were evaluated using 95% credible intervals for the involved parameters and models were compared using the Deviance Information Criterion (DIC) [29] which is readily computed from the MCMC runs. The DIC is a model evaluation criterion, which, analogous to the AIC (Akaike Information Criterion) and BIC (Bayesian Information Criterion) criteria, combines a measure of model fit with a penalty for model complexity. The DIC is defined as *DIC *= -2 log(likelihood) +2 · *ρ*_*D*_, where the first term is also known as the deviance and the second term increases with the effective number of model parameters. Both the deviance and the *p*_*D *_are estimated during the MCMC run. When comparing two models, the model with smaller DIC is usually preferred. For visual model comparison the posterior mean and credible intervals were also computed for *λ *= *β*_0_*κ *and $\lambda =\beta_{0}\kappa^{\beta_{1}}$ for increasing values of *κ *for models 1 and 2, respectively. R^2 ^values were computed as R^2 ^= 1-SS_E_/SS_Tot_, where SSE and SSTot are the error sum of squares and total corrected sum of squares for LTC counts, respectively.

In a similar manner, posterior means and 95% credible intervals of exp *θ*_*i *_and exp $\theta_{i}^{\beta_{1}}$ were computed for the month effects in model 1 and model 2, respectively. These numbers give the multiplicative effect of month to the expected counts for both *y*_*ijk *_and *x*_*ijk*_. In these computations the month of July was chosen as a base level for which *θ*_*3 *_*= 0*, hence, all other month effects are relative to July. Then exp *θ*_*1 *_will be the factor of change in expected counts when comparing March counts with July counts, and so on. Correspondingly, house 1 was chosen as the base level house with *γ*_*1 *_= 0, and posterior means and 95% intervals were computed for the multiplicative house effects exp *γ*_*j *_and exp $\gamma_{i}^{\beta_{1}}$ for model 1 and 2, respectively.

Pearson's chi-square analysis was used to test if the number of blood-fed mosquitoes collected, either indoors or outdoors, was associated with the method of collection.

Raw data of collected and analyzed mosquitoes are provided as supplementary files (Additional file 1 and Additional file 2).

## Results

A total of 12,999 *Anopheles *mosquitoes were collected throughout the study period (Table 1). The total number of *Anopheles *collected indoors by human landing catches and in light traps was 4,939 (84%) and 914 (16%), respectively. The corresponding numbers for the outdoor collections were 6,883 (96%) and 263 (4%). *Anopheles gambiae *senso stricto was the predominant species in Mongola (99.7% of successfully identified specimens). In Arena Blanca, *Anopheles melas *was the most common species (92.0%), and in Riaba both *An. gambiae *and *An. melas *were prevalent (46.1% *An. gambiae *s.s and 53.9% *An. melas*). Only 13 (5.1%) of the 256 HLCs (outdoor and indoor man-nights combined) yielded no mosquitoes, none of which occurred in Mongola; nine occurred (11.2%) in Arena Blanca; and four occurred (5.0%) in Riaba. Ten of the 13 zero catches were from the indoor collections. On the other hand, as many as 130 (50.8%) of the 256 LTC sampling occasions (outdoor and indoor light trap catches combined) did not yield any mosquitoes; 47 (49.0%) in Mongola, 32 (40.0%) in Arena Blanca, and 51 (63.8%) in Riaba. About 62% of the LTC zero catches were from the outdoor collections. The proportion of blood-fed mosquitoes was generally higher indoors than outdoors, particularly for the LTC (Table 1). The chi-square analysis showed that the number of blood fed mosquitoes collected indoors was associated with the collection method (*χ*^2 ^= 4,15; df = 1, *p *= 0,0416), whereas in the outdoor collections it was independent of the collection method (*χ*^2 ^= 1,1; df = 1, *p *= 0,2943).

**Table 1:** Anopheline mosquitoes collected by human landing (HLC) and light trap collections (LTC) indoors and outdoors, number of blood-fed mosquitoes, and number of *Anopheles gambiae *s.l. identified to species in Mongola, Arena Blanca and Riaba, Bioko Island, Equatorial Guinea in 2009

	Mongola		Arena Blanca		Riaba		
	**HLC**	**LTC**	**HLC**	**LTC**	**HLC**	**LTC**	**Sum**

Total number of Anophelines collected	7604	429	2905	629	1313	119	12999

Indoors	3172	391	1270	460	497	63	5853

Numbers blood fed (%)	531	106	258	66	128	24	1113
	
	(16.7)	(27.1)	(20.3)	(14.3)	(25.8)	(38.1)	(19.0)

Outdoors	4432	38	1635	169	816	56	7146

Numbers blood fed (%)	707	6	346	19	197	16	1291
	
	(16.0)	(15.8)	(21.2)	(11.2)	(24.1)	(28.6)	(18.1)

Total number of *Anopheles gambiae *s.l. identified	1230	332	929	512	918	86	4007

*An. gambiae *s.s.	1194	330	73	19	422	16	2054

*An. melas*	4	2	844	486	493	70	1899

Identification failure*	32	0	12	7	3	0	54

* Possible reasons for failure of molecular identification could be pipetting error or morphological misidentification, i.e. the specimen did not belong to the *An. gambiae *complex

**Figure 2:**
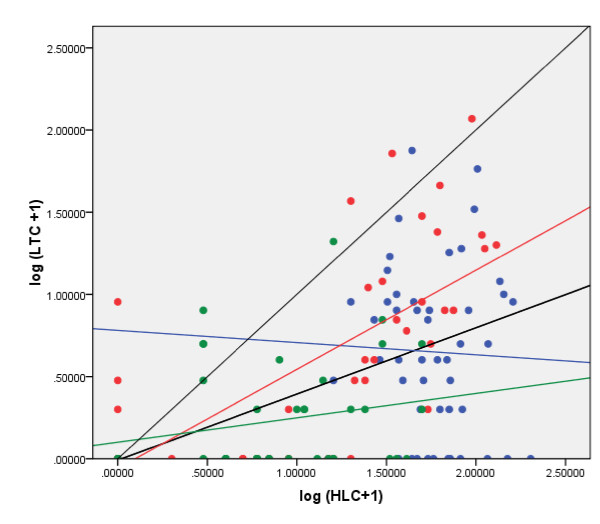
**Relationships between human landing and light trap collections of *Anopheles gambiae *mosquitoes, Bioko Island, 2009**. Regression lines show all sites together (thick black), Mongola (blue), Arena Blanca (red), Riaba (green). The thin black line indicates the line of identity

**Figure 3:**
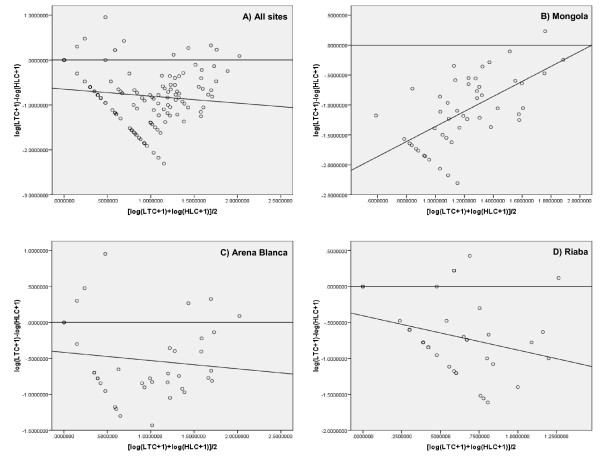
**Relationships between relative sampling efficiency of indoor light traps and mosquito abundance, Bioko Island, 2009**. The relative sampling efficiency of light traps is the difference in mosquitoes collected indoors by light trap and human landing collections (y-axis). The mosquito abundance is the joint average number of mosquitoes (x-axis). The relationship is shown for A) all sites, B) Mongola, C) Arena Blanca, and D) Riaba

Analysis of the entire indoor data set (n = 128) showed a significant positive correlation (r = 0.45; *p *< 0.0001) between the two collection methods (Table 2, Figure 2). When analysed by location, the correlation was also significant for Arena Blanca (r = 0.66; *p *< 0.0001), but not for Mongola and Riaba. Based on non-log transformed correlations with intercept set at zero, the calculated LTC:HLC ratios varied between 0.07-0.34 for the indoor collections and 0.01-0.09 for the outdoor collections. However, these ratios are highly uncertain since the usual model assumption of normal distributed errors with constant variance is violated, the R^2 ^values are low (R^2 ^= 0.07-0.40), and there are likely non-linear relationships between the counts (see Bayesian approach below). The relative sampling efficiencies were plotted against mosquito abundance for all sites and for each site separately (Figure 3). Only in Mongola was the regression slope significantly different from zero (*p *< 0.0001) (Table 2), meaning that the relative sampling efficiency was dependent on mosquito density in this site; i.e. as mosquito density increases so does the LTC:HLC ratio. This was not seen in the other sites, or all sites together, where the relative sampling efficiency was independent of mosquito density.

**Table 2:** Correlation and regression analysis of log-transformed indoor human landing (HLC) and light trap (LTC) collections of *Anopheles gambiae *s.l. on Bioko Island, 2009. The correlation coefficients show the relationship between *log(LTC + 1) *and *log(HLC + 1)*). The regression slopes are from regressing relative sampling efficiencies (*log(LTC + 1)-log(HLC + 1)*) on average abundance (*[log(LTC + 1) + log(HLC + 1)]/2*)

		Correlation coefficient			Regression slope		
**Site**	**n**	**r**	***p***	**b**	**95% C.I**.	**t**	***P***

All	128	0.451	< 0.0001	-0.155	-0.371-0.061	-1.424	0.157

Mongola	48	-0.037	0.80	1.255	0.770-1.739	5.213	< 0.0001

Arena Blanca	40	0.663	< 0.0001	-0.114	-0.409-0.181	-0.785	0.438

Riaba	40	0.199	0.22	-0.478	-0.996-0.040	-1.867	0.07

n = sample size, r = Pearson's correlation coefficient, b = regression slope, C.I. = confidence interval, t = *t*-test value, *p *= probability value

**Figure 4:**
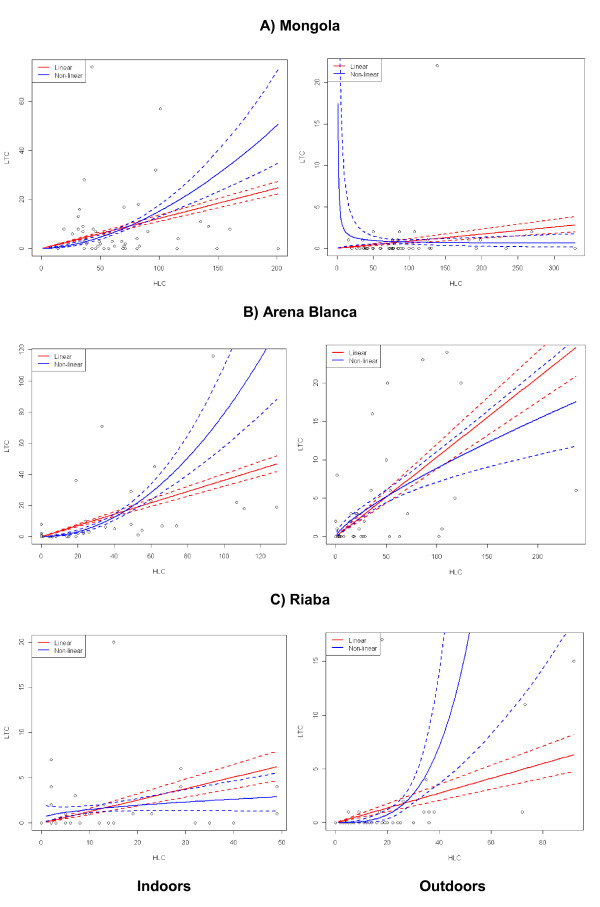
**Relationships between light trap collections and human landing collections using Bayesian analysis**. Light trap collections (LTC) *versus *human landing collections (HLC) counts for indoor (left panel) and outdoor (right panel) counts for A) Mongola, B) Arena Blanca, and C) Riaba. Estimated expected counts for LTC (with 95% credible interval bands) are given according to model 1 (red) and model 2 (blue)

When applying the Bayesian approach, the non-linear model provided a better fit to the indoor data for all locations, particularly for Mongola and Arena Blanca, because the DIC values are smaller for the non-linear than for the linear model (Table 3). For these two locations the 95% credible intervals of *β*_*1 *_do not include the unit value (*β*_*1 *_= 1), representing model equality (Table 3), also visually verified in Figure 4 (top two left graphs) showing a clear separation between the two models and their credible bands. Hence, for Mongola and Arena Blanca the LTC and HLC counts appeared to be non-proportional, implying that the ratio of LTC to HLC counts is density dependent and a straightforward conversion factor between HLC and LTC counts cannot be calculated. For Riaba, the difference between the two models is minimal, but also here the non-linear model has a slightly better fit than the linear model (Table 3). For Riaba the value of *β*_*1 *_is significantly smaller than one indicating that the LTC:HLC ratio decreases with density, whereas for the two other sites the value of *β*_*1 *_was larger than one yielding an increasing LTC:HLC ratio with density (Table 3). The fact that the credible interval for *β*_*1 *_for Riaba covers zero indicates that it cannot be excluded that the expected LTC count is independent of the expected HLC count for this site. This is also verified by the fact that the credible bands for the non-linear model are not in conflict with a true horizontal curve (lower left graph in Figure 4). The weak non-linearity and possible absence of association between the expected LTC and HLC counts suggest that a conversion factor between the indoor counts cannot be computed for Riaba. Generally, the R^2 ^values of the models were very low, particularly of those outdoors and those in Riaba (Table 3).

**Table 3:** Summary statistics from model estimates.

Site	Model	Indoor		Outdoor	
		
	parameters	Model 1	Model 2	Model 1	Model 2
		0.1231	0.0082	0.0085	17.4800
		
Mongola	${\hat{\beta }}_{0}$	(0.1108- 0.1364)	(0.0012- 0.0280)	(0.0060- 0.0116)	(0.0572 - 130.9)
	
	${\hat{\beta }}_{1}$	-	1.702	-	-0.2934
		
			(1.3470- 2.0770)		(-1.1760- 0.5901)
	
	DIC	717.318	680.630	206.033	199.661
	
	R^2^	0.124	0.172	-0.0175	-0.00197

Arena Blanca	${\hat{\beta }}_{0}$	0.3624	0.0104	0.1034	0.2873
		
		(0.3239- 0.4030)	(0.0020-0.0337)	(0.0878- 0.1204)	(0.0969-0.6085)
	
	${\hat{\beta }}_{1}$	-	1.9930	-	0.7673
		
			(1.6250- 2.3570)		(0.5485 - 1.013)
	
	DIC	722.166	665.462	352.945	355.456
	
	R^2^	0.258	0.342	0.0842	0.107

Riaba	${\hat{\beta }}_{0}$	0.127	0.7376	0.0688	0.0005
		
		(0.0954- 0.1621)	(0.1742- 1.9140)	(0.0519- 0.0890)	(1.808E-7- 0.0039)
	
	${\hat{\beta }}_{1}$	-	0.3793	-	3.191
		
			(-0.0727- 0.8747)		(1.8810 - 4.8940)
	
	DIC	216.482	215.631	203.796	215.631
	
	R^2^	0.00136	0.0123	0.0815	0.116

For each model and parameter the posterior mean is given with 95% credible intervals (in parenthesis). Model 1 indicates proportionality (linear) and model 2 density dependence (non-linear) between light trap collections and human landing collections. A smaller Deviance Information Criterion (DIC) indicates a better fit when comparing models (further details in text)

For the outdoor data, the linear relations between expected LTC and HLC counts show better fit, particularly for Arena Blanca and Riaba. For Arena Blanca the non-linearity is minimal and non-significant in the sense that one is included in the credible interval for *β*_*1*_, meaning the two models are equivalent resulting in the best fitted linear model of all locations. The conversion factor estimate for Arena Blanca is ${\hat{\beta }}_{0}=0.1034$. For Riaba the linear model has the lower DIC even though the credible interval for *β*_*1 *_for the non-linear model is entirely above one, but this is probably mostly due to a single observation for which LTC = 18 and HLC = 17. The conversion factor estimate for Riaba is ${\hat{\beta }}_{0}=0.0688$. In Mongola, the model estimates in Figure 4 (top right panel) indicate that the non-linear model fit is not good, and the fact that this model has a lower DIC than the linear model merely indicates that neither the linear nor the non-linear model fit should be trusted in this case. Even though a linear model may appear significant, consistency of the conversion factor between LTC and HLC is absent. Presenting an outdoor conversion factor for Mongola is therefore pointless.

**Figure 5:**
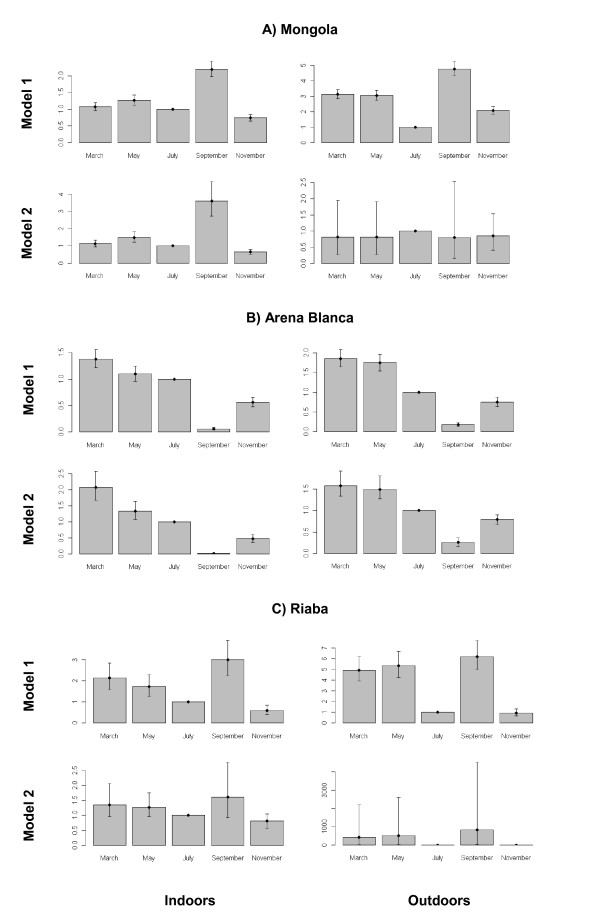
**Monthly effects on expected light trap collections using Bayesian analysis**. Posterior means and 95% credible intervals for monthly effects on mosquitoes collected indoors (left panels) and outdoors (right panels) in A) Mongola, B) Arena Blanca, and C) Riaba using linear (model 1, upper panels) and non-linear models (model 2, lower panels). July is the reference month.

There are large monthly variations in expected counts based on the outcomes of the two models. For instance, in Mongola the expected indoor LTC counts (hence also HLC counts since these depend on the expected LTC counts) are estimated to be two to three times higher in September than in July (model 1 and 2, Figure 5a, left panels). The same pattern in month effects is seen in Riaba (model 1 and 2, Figure 5c, left panels). For Arena Blanca the results are rather different. Both models and both indoor and outdoor counts show the lowest expected LTC counts for September with a steadily decrease through the year. The results from Arena Blanca appear to be the most consistent across both venue and model. The least reliable results are the outdoor effects in Mongola and Riaba where the 95% credible intervals are very wide indicating high variance. In general the higher flexibility of model 2 also increases the uncertainty of the month effects.

Posterior means and 95% credible intervals for monthly effects on mosquitoes collected indoors (left panels) and outdoors (right panels) in A) Mongola, B) Arena Blanca, and C) Riaba using linear (model 1, upper panels) and non-linear models (model 2, lower panels). July is the reference month

There are also large variations between houses (results not shown). The house effect may be considered a random nuisance effect in a classical statistical manner, which is important to include in the model to correct for the extra variability this factor brings to the observed counts.

## Discussion

The feasibility of determining reliable conversion factors between light trap mosquito collections (LTC) and human landing mosquito collections (HLC) was evaluated on Bioko Island, Equatorial Guinea. The ultimate aim was to examine if light traps suspended indoors adjacent to occupied long-lasting, insecticide-treated bed nets can provide reliable estimates of human biting rates for entomological surveillance in the BIMCP. The results, based on analyses by both simple regression analysis and Bayesian statistical approaches [14,26], indicate that reliable conversion factors between LTC and HLC could not be calculated for Bioko Island.

As far as the indoor collections are concerned, the results from this study indicate that in Arena Blanca, where 27% of the total number of mosquitoes were collected and 92% of the identified specimens were *An. melas*, the correlation between indoor collection methods was highest (r = 0.66), which is comparable to other studies on *An. gambiae *s.l. [11,12]. Although the simple regression analyses indicated that the relative sampling efficiency was unaffected by mosquito density, the more robust Bayesian analysis showed a non-linear relationship between collection methods in Arena Blanca. This means that the relative sampling efficiency is density dependent and a simple conversion factor between indoor LTC and HLC counts cannot be calculated. In Mongola, where 62% of all anophelines were collected and close to 100% were identified as *An. gambiae *s.s., there was no apparent correlation between collection methods. Both statistical analyses showed that the relative sampling efficiency was dependent on mosquito density, again implying that conversion factors could not be calculated. In Riaba, where only 11% of the total number of mosquitoes were collected and both *An. gambiae *s.s. and *An. melas *occurred in nearly equal proportions, there was no apparent correlation between methods carried out indoors. The Bayesian analyses indicated, at most, a weak non-linear but possibly non-existent relationship between the expected LTC and HLC counts and no conversion factor could be calculated. Furthermore, the Bayesian models could only explain 35% or less of the variation (R^2^-values), which again substantiates the poor fit of these models. For example, the R^2 ^values for the outdoor collections in Mongola were, in fact, negative, which indicates a poorer fit than a model simply based on an average. In other words no, or even misleading, information is provided by these models.

Comparisons of different mosquito collection methods have shown results to vary by mosquito density [20,21], mosquito species [7,14], external stimuli, such as urban illumination [19], availability of alternative hosts [16], or various unknown factors [18]. In addition, the present study indicates that the statistical method used to analyse data will affect results. On Bioko Island there was a poor correlation between the two collection methods (Table 2) and results varied by many factors, such as location, venue, month, and collection point (house). The two statistical methods used here show inconsistent results, suggesting that simple correlations between absolute non-transformed mosquito counts from different collection methods, as performed in some studies [7], do not provide reliable results. Furthermore, the log (x + 1) transformation, commonly used with the Altman and Bland method [26] to compare different mosquito collection methods [e.g. [9,12,21]], does not approximate log (x) [23] and, therefore, may give different results depending on mosquito density. Analyses of trap comparison data should preferably use model estimation methods, such as Bayesian parameter estimation, assuming Poisson or negative binomially distributed data [13-16]. The fact that different statistical methods convey inconsistent results, as presented here, is a strong indication that results are highly questionable and that there is very weak evidence of relationships in the data.

Outdoor biting of *An. gambiae *s.l. is a common characteristic on Bioko Island [30] and outdoor conversion factors would be operationally highly relevant. In the present study, the Bayesian analyses of the outdoor collections generally indicate linear relationships between LTC and HLC. The best fitted linear models were found for Arena Blanca and Riaba, with outdoor LTC:HLC conversion factors of 0.10 and 0.07, respectively, meaning that one mosquito in a light trap would correspond to approximately 10 mosquitoes collected by human landing in Arena Blanca (likely an *An. melas*) and 14 mosquitoes in Riaba. Although it can be argued that these factors may be valid for these specific sites, their operational usefulness for entomological monitoring by the BIMCP is doubtful for several reasons. The calculated coefficients of determination (R^2 ^< 0.40) are quite low, leaving a large proportion of variability unexplained, thus indicating that the predictions of HLC from LTC counts is highly unreliable. Further, other sites on the island may show other relationships and the failure of finding a relationship in Mongola, in addition to these poor results, indicate that the prospect of finding reliable conversion factors is not very promising. The predominance of *An. melas *in Arena Blanca may not be the same for another year's collection and the relative contribution of the two different species collected in Riaba to these results cannot be established based on these analyses. Furthermore, the effectiveness of outdoor light trap collections is highly variable depending on many factors, such as mosquito species, trap location, weather conditions, etc. [6]. The absence of a human bait for the outdoor traps will affect sampling efficiency, as anthropophilic mosquitoes, such as *An. gambiae *s.l., primarily respond to host cues rather than a light source [11]. Moreover, light traps placed outdoors are more exposed to adverse weather conditions than indoor traps. For example, the likely reason for the low mosquito numbers collected in September 2009 in Arena Blanca (Figure 5) was strong prevailing winds at the time of collection.

The conversion factors for Arena Blanca and Riaba, although calculated from outdoor collections, are among the lowest reported in Africa [9-13,15-18] and approach the extremely low light trap collection efficiency (LTC:HLC = 0.02) found in urban Dar es Salaam, Tanzania [19]. The authors of that study suggested that the poor light trap performance was affected by the city lights and concluded that light traps are not appropriate for mosquito surveillance and monitoring the impact of mosquito control measures in Dar es Salaam [19]. The illumination hypothesis is not very likely for explaining the poor performance of light traps on Bioko Island. Evidently light traps also perform poorly in a sparsely populated, humid tropical environment such as Bioko Island.

In Mongola and Arena Blanca, the collected species consisted of almost 100% *An. gambiae *s.s. and *An. melas*, respectively. Therefore, these sites might be expected to give potentially the most meaningful results, because it is likely that each species has its own specific collection pattern. However, it is not clear from the present data if the observed differences reflect species-specific or location-specific variation. Furthermore, species prevalence may be affected by inter- and intra-annual variation, weather conditions, sampling error, and/or other stochastic factors.

It is interesting to note that quite many blood-fed mosquitoes were collected in light traps, particularly in the indoor collections. Consequently, in this setting, light traps could be a fairly good tool to monitor mosquito sporozoite rates. However, human biting rates estimated by human landing catches are still needed to calculate entomological inoculation rates. The relatively higher proportion of blood-fed mosquitoes indoors likely reflects the fact that after a blood meal is taken, these mosquitoes are simply looking for a resting spot and if there is a light trap nearby, they could be preferentially attracted to the trap rather than exiting the house. In absolute numbers there were more blood feds in the HLC collections which probably is a result of mosquitoes feeding on the collectors at the time of collecting.

In many similar studies insecticide-free bed nets were used as it was thought that treated nets might repel mosquitoes and bias results. However, in Zambia no difference was found in the number of *An. arabiensis *collected in CDC light traps suspended next to people sleeping under a deltamethrin-treated net *versus *untreated nets [13]. Furthermore, Magbity *et al*. [12] found only a slight reduction in the relative sampling efficiency of light traps in villages where people slept under lambdacyhalothin-treated nets compared to villages with no nets. In Dar es Salaam there was no significant difference between the proportion of *An. gambiae *s.l. caught indoors in houses with long-lasting, insecticide-treated nets (LLINs) *versus *houses with untreated bed nets [19]. Any attempt to estimate practical and operational conversion factors should use LLINs as it is now the most commonly used personal protection method for malaria control.

This study investigated one component of the entomological inoculation rate, the abundance of host-seeking mosquitoes, which is a proxy of the human biting rate. Data on trap-specific variations in sporozoite rates are needed to estimate the effect of collection method on the entomological inoculation rate. Furthermore, it is not possible, from these data, to assess variations in parity and age composition to evaluate whether the methods sampled different fractions of mosquito populations. If this study had resulted in a consistent and operationally relevant conversion factor between the two collection methods, these issues would need to be investigated. Because mosquitoes of different parity and age will have different sporozoite rates and thus ability to infect people, such a difference between trapping methods would bias EIR estimates, even with a reliable conversion factor.

## Conclusions

These results do not provide support for a reliable conversion factor between light traps and human landing collections for Bioko Island. Relationships between catches using the two methods appear, in general, to be non-linear. In addition, the results also depended on the statistical methods used, indicating a lack of robustness. Even in a relatively small and confined area such as Bioko Island, the dynamics of mosquito catches vary depending on site, time of the year, species composition, and stochastic factors, such as weather conditions, etc. A practical, realistic, and operationally feasible mosquito collection method for establishing human biting rates should not be fraught with inconsistencies depending on factors such as mosquito density, which may vary from month to month, or which is applicable only to some mosquito species and not to others. Based on data presented here, light trap collections are not recommended as a method to assess human biting rates. Therefore, despite the potential ethical implications of exposing human volunteers to potentially infectious mosquito bites, controlled human landing collections with well-trained and consenting collectors, good field supervision, compulsory health follow-ups and accessible malaria treatment of collectors remain, for the time being, the only way to determine realistic human biting rates.

## Competing interests

The authors declare that they have no competing interests.

## Authors' contributions

HJO conceived, supervised, and planned the study, supervised, performed field collections and wrote the first draft manuscript. SS performed the Bayesian statistical analysis, wrote parts of the statistical methods and result sections, and provided general statistical advice. MRR participated in the study design and provided editorial input. VPR conducted molecular analyses. SA participated in the study design, supervised and participated in field collections, and provided editorial input. AM participated in study design, participated in field collections, and provided editorial input. MAS participated in study design, supervised the molecular analysis, and contributed to manuscript preparation. All authors read and approved the final manuscript.

## Supplementary Material

Additional file 1 **Number of Anopheline mosquitoes and proportion blood-fed collected on Bioko Island, Equatorial Guinea, 2009**. Click here for file

Additional file 2 ***Anopheles gambiae *s.l. mosquitoes identified to species and molecular form from mosquito collections on Bioko Island, Equatorial Guinea, 2009**. Click here for file
